# Supplementary material for: Cranberry Proanthocyanidins as a Therapeutic Strategy to Curb Metabolic Syndrome and Fatty Liver-Associated Disorders
Source: Antioxidants (Basel). 2022 Dec 30;12(1):90. doi: 10.3390/antiox12010090 (PMC9854780; doi:10.3390/antiox12010090)
Supplement: Supplementary file 1 [file antioxidants-12-00090-s001.zip › antioxidants-2035530-supplementary ú¿Table S1ú⌐.pdf]

Table S1. Cranberry PAC polymeric fraction characterization

Analyte	Concentration (mg/g)
Total proanthocyanidins *	505 ± 12
Mean DP *	6.82 ± 0.03
Procyanidins A2	2.08 ± 0.006
Trimeric PAC (1 and 2 A-type bounds)	4.93 ± 0.12
Procyanidins B	0.81 ± 0.02
Flavan-3-ols total	1.29 ± 0.03
Flavonols total	0,13 ± 0.01
Total anthocyanins	0.21 ± 0,01

*Determined by phloroglucinolysis; DP, Degree of polymerization
