# Supplementary material for: Cranberry Proanthocyanidins as a Therapeutic Strategy to Curb Metabolic Syndrome and Fatty Liver-Associated Disorders
Source: Antioxidants (Basel). 2022 Dec 30;12(1):90. doi: 10.3390/antiox12010090 (PMC9854780; doi:10.3390/antiox12010090)
Supplement: Supplementary file 1 [file antioxidants-12-00090-s001.zip › supplementary data_12-12-2022.pptx]

## Slide 1
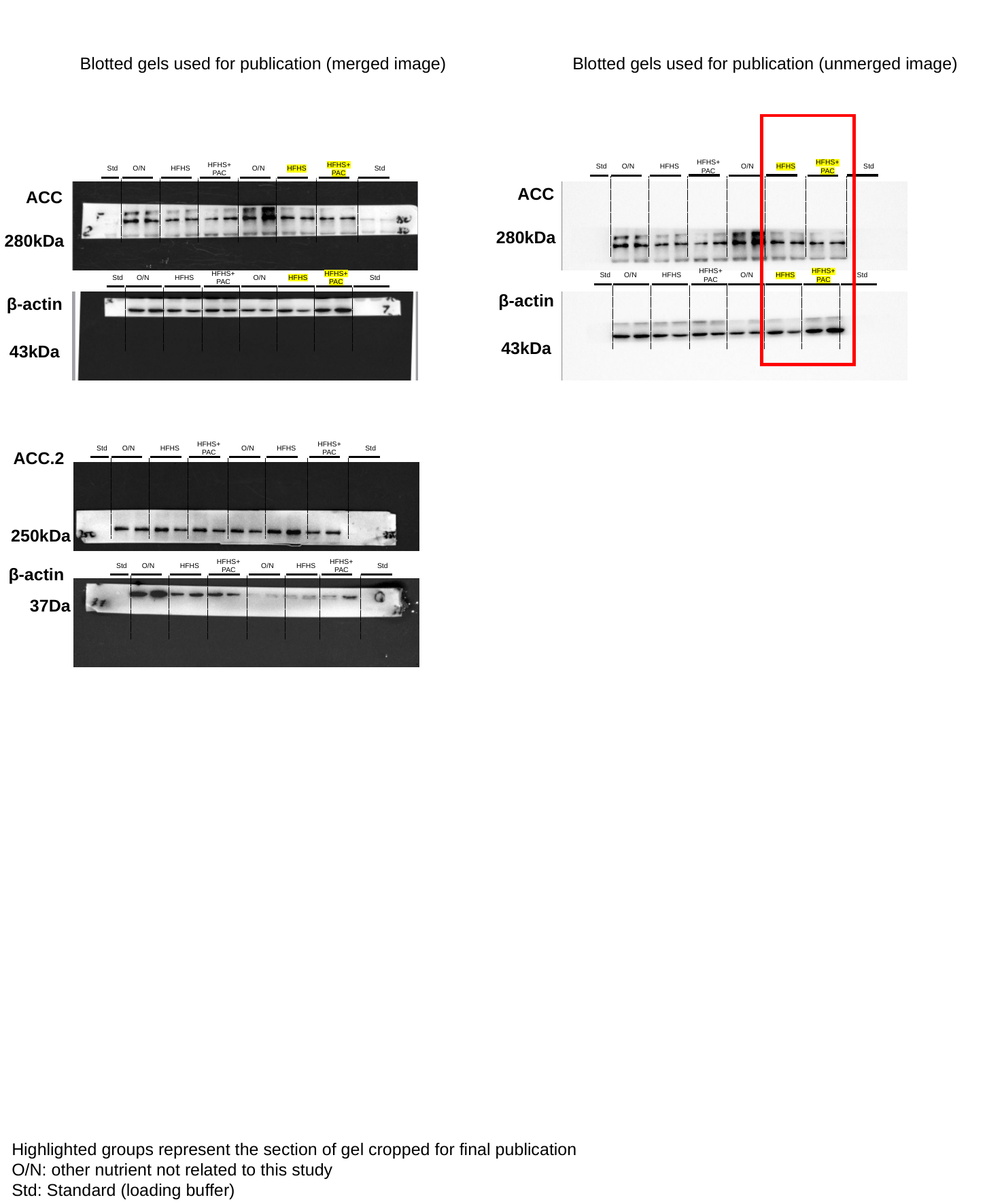

Blotted gels used for publication (merged image)
Blotted gels used for publication (unmerged image)
HFHS+PAC
HFHS+PAC
Std
O/N
HFHS
O/N
HFHS
Std
HFHS+PAC
HFHS+PAC
Std
O/N
HFHS
O/N
HFHS
Std
ACC
280kDa
43kDa
β-actin
ACC
280kDa
43kDa
HFHS+PAC
HFHS+PAC
Std
O/N
HFHS
O/N
HFHS
Std
HFHS+PAC
HFHS+PAC
Std
O/N
HFHS
O/N
HFHS
Std
β-actin
HFHS+PAC
Std
O/N
HFHS
O/N
HFHS
HFHS+PAC
Std
ACC.2
β-actin
250kDa
HFHS+PAC
HFHS+PAC
Std
O/N
HFHS
O/N
HFHS
Std
37Da
Highlighted groups represent the section of gel cropped for final publication
O/N: other nutrient not related to this study
Std: Standard (loading buffer)

## Slide 2
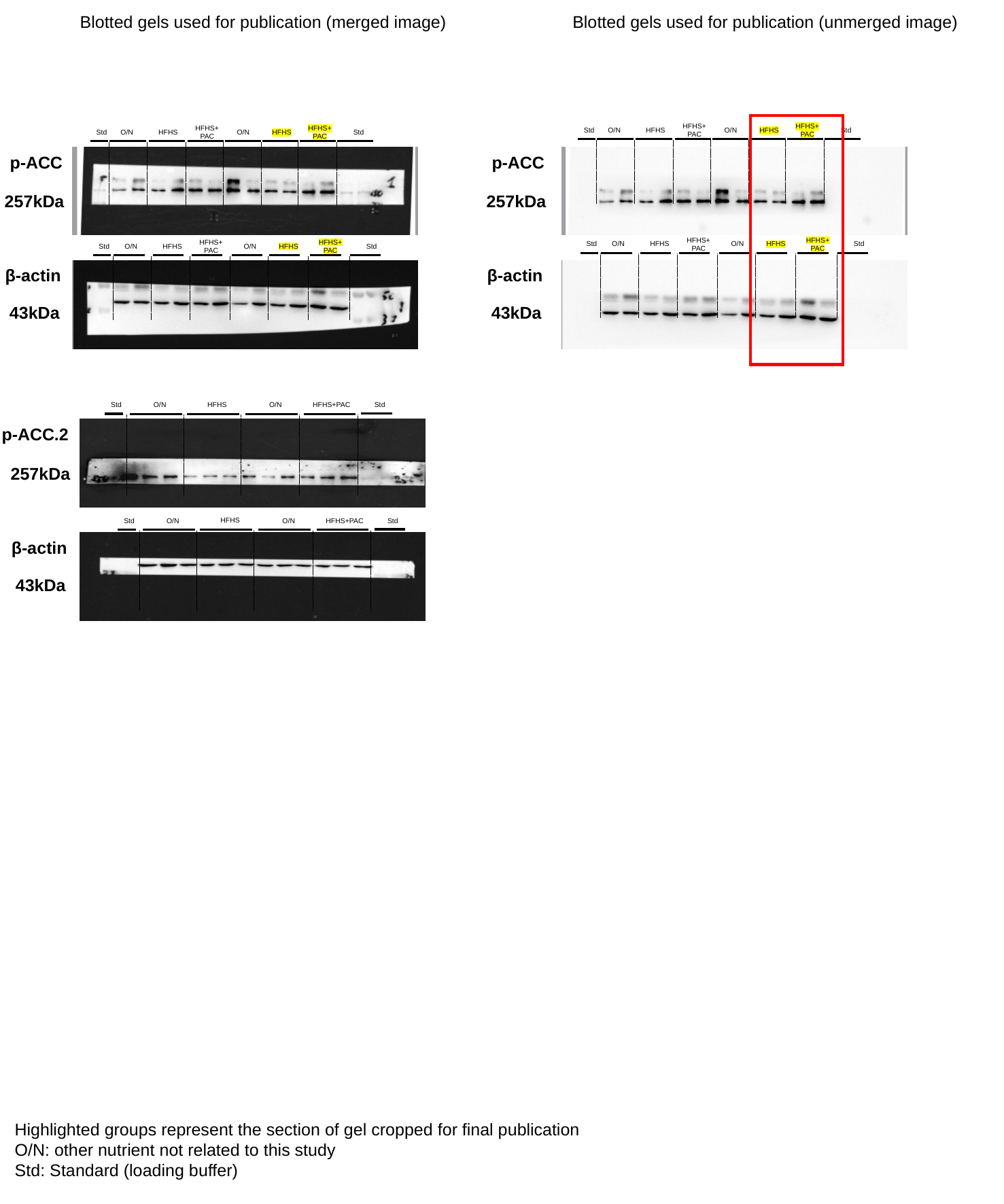

Blotted gels used for publication (merged image)
Blotted gels used for publication (unmerged image)
HFHS+PAC
HFHS+PAC
Std
O/N
HFHS
O/N
HFHS
Std
HFHS+PAC
HFHS+PAC
Std
O/N
HFHS
O/N
HFHS
Std
p-ACC
257kDa
β-actin
43kDa
p-ACC
257kDa
β-actin
43kDa
HFHS+PAC
HFHS+PAC
Std
O/N
HFHS
O/N
HFHS
Std
HFHS+PAC
HFHS+PAC
Std
O/N
HFHS
O/N
HFHS
Std
HFHS
Std
O/N
Std
O/N
HFHS+PAC
p-ACC.2
257kDa
β-actin
43kDa
HFHS
Std
O/N
Std
O/N
HFHS+PAC
Highlighted groups represent the section of gel cropped for final publication
O/N: other nutrient not related to this study
Std: Standard (loading buffer)

## Slide 3
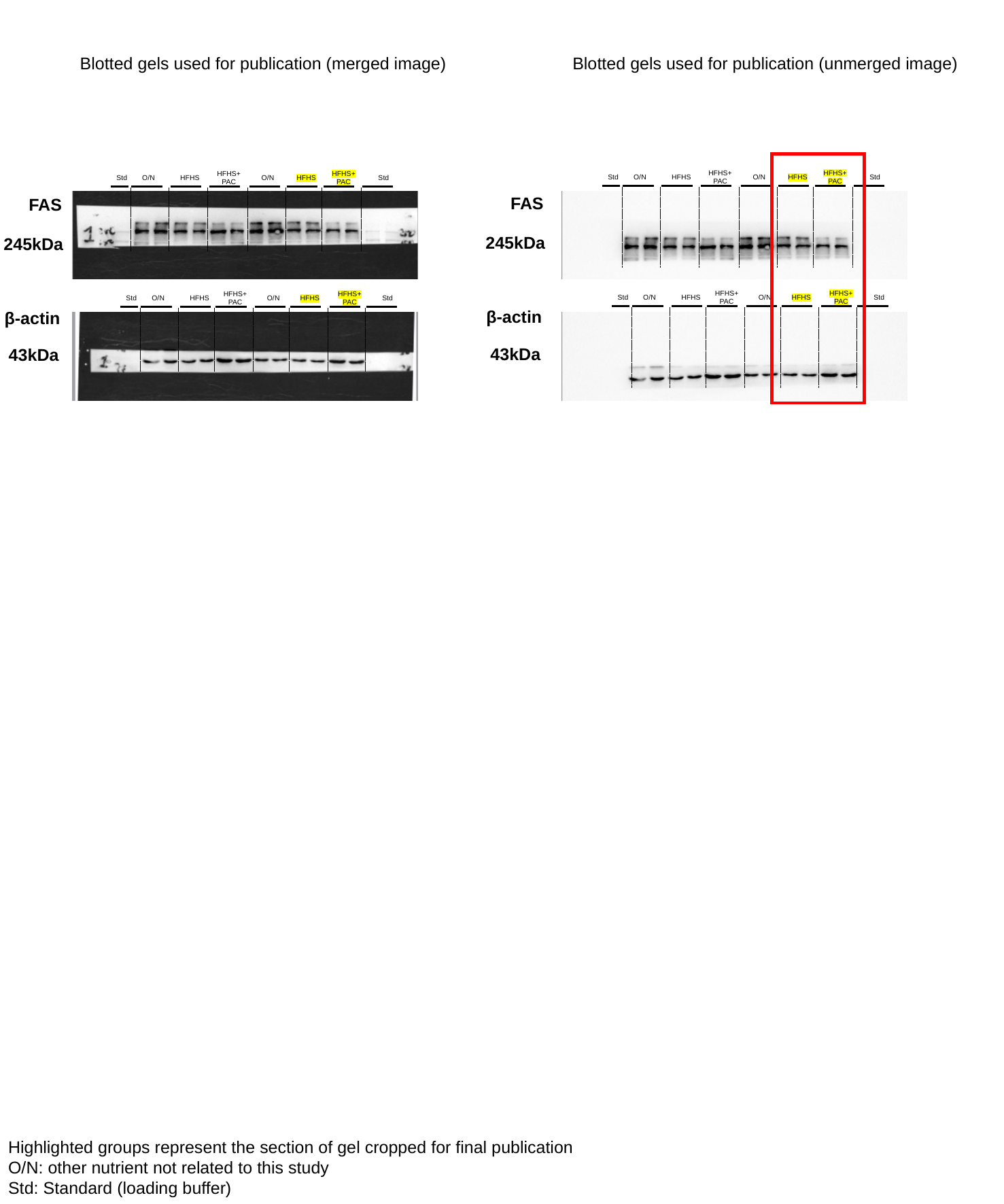

Blotted gels used for publication (merged image)
Blotted gels used for publication (unmerged image)
HFHS+PAC
HFHS+PAC
Std
O/N
HFHS
O/N
HFHS
Std
HFHS+PAC
HFHS+PAC
Std
O/N
HFHS
O/N
HFHS
Std
FAS
245kDa
β-actin
43kDa
FAS
245kDa
β-actin
43kDa
HFHS+PAC
HFHS+PAC
Std
O/N
HFHS
O/N
HFHS
Std
HFHS+PAC
HFHS+PAC
Std
O/N
HFHS
O/N
HFHS
Std
Highlighted groups represent the section of gel cropped for final publication
O/N: other nutrient not related to this study
Std: Standard (loading buffer)

## Slide 4
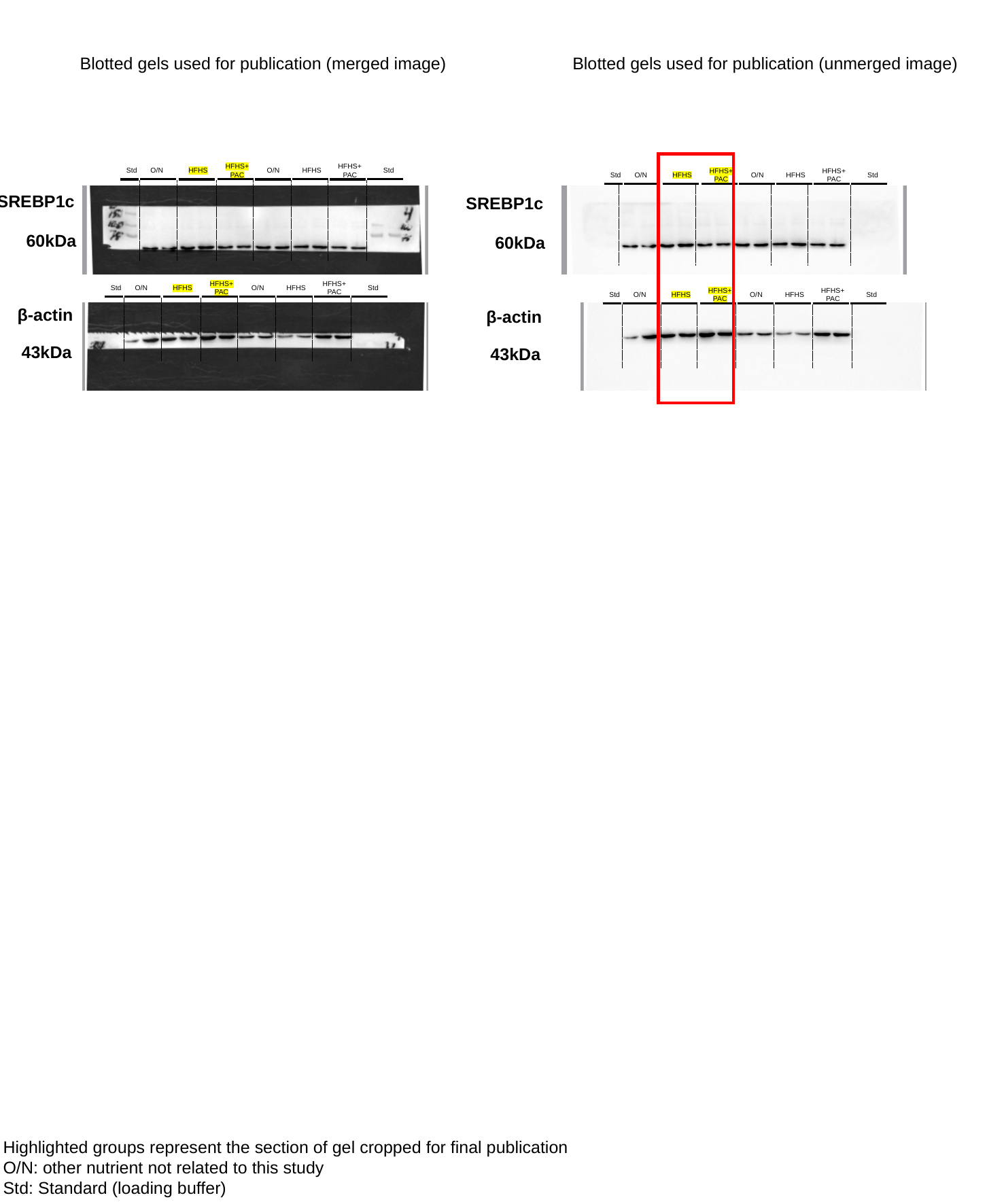

Blotted gels used for publication (merged image)
Blotted gels used for publication (unmerged image)
HFHS+PAC
HFHS+PAC
Std
O/N
HFHS
O/N
HFHS
Std
HFHS+PAC
HFHS+PAC
Std
O/N
HFHS
O/N
HFHS
Std
SREBP1c
60kDa
β-actin
43kDa
SREBP1c
60kDa
β-actin
43kDa
HFHS+PAC
HFHS+PAC
Std
O/N
HFHS
O/N
HFHS
Std
HFHS+PAC
HFHS+PAC
Std
O/N
HFHS
O/N
HFHS
Std
Highlighted groups represent the section of gel cropped for final publication
O/N: other nutrient not related to this study
Std: Standard (loading buffer)

## Slide 5
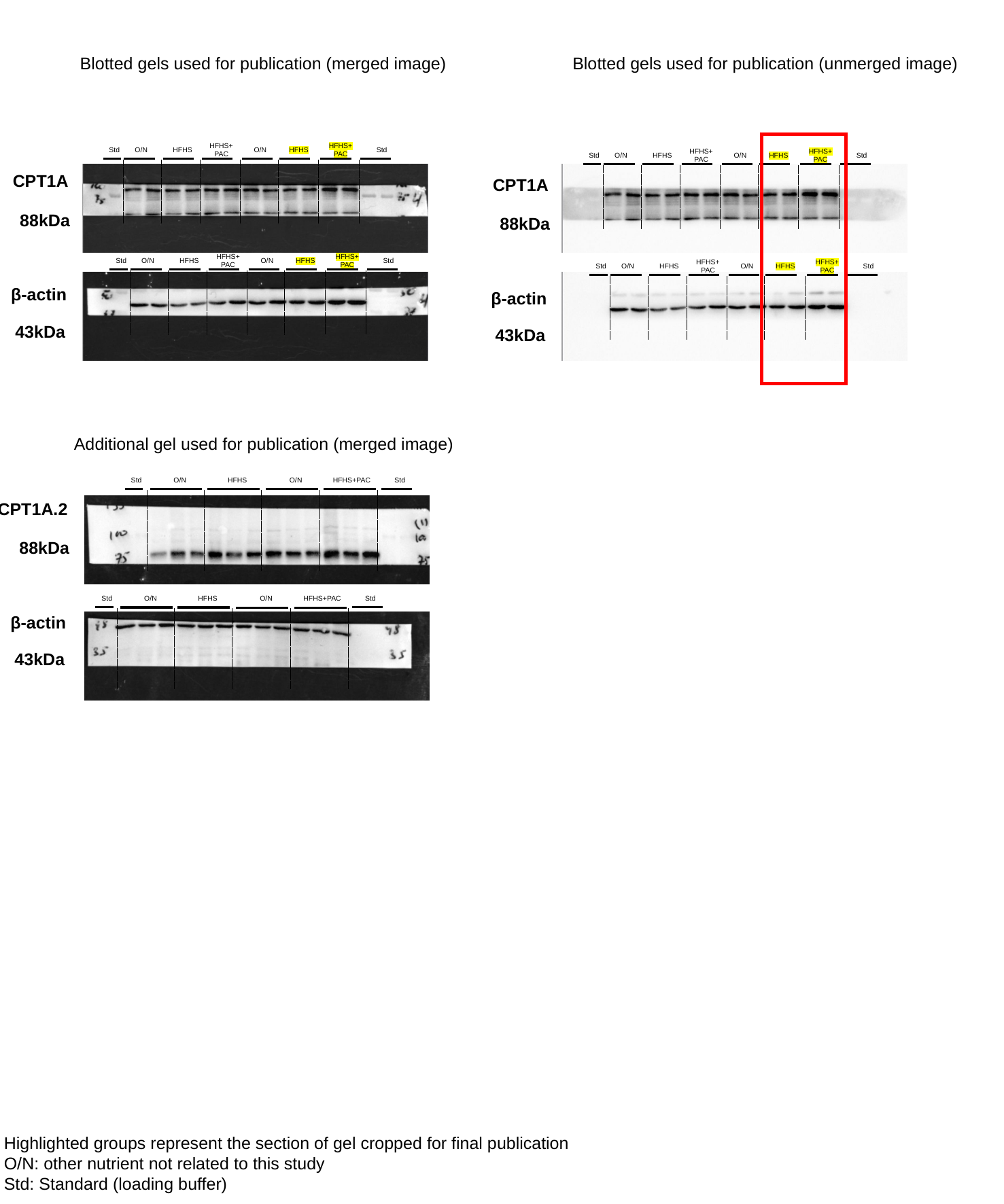

Blotted gels used for publication (merged image)
Blotted gels used for publication (unmerged image)
HFHS+PAC
HFHS+PAC
Std
O/N
HFHS
O/N
HFHS
Std
HFHS+PAC
HFHS+PAC
Std
O/N
HFHS
O/N
HFHS
Std
CPT1A
88kDa
β-actin
43kDa
CPT1A
88kDa
β-actin
43kDa
HFHS+PAC
HFHS+PAC
Std
O/N
HFHS
O/N
HFHS
Std
HFHS+PAC
HFHS+PAC
Std
O/N
HFHS
O/N
HFHS
Std
Additional gel used for publication (merged image)
HFHS
Std
O/N
Std
O/N
HFHS+PAC
HFHS
Std
O/N
Std
O/N
HFHS+PAC
CPT1A.2
88kDa
β-actin
43kDa
Highlighted groups represent the section of gel cropped for final publication
O/N: other nutrient not related to this study
Std: Standard (loading buffer)

## Slide 6
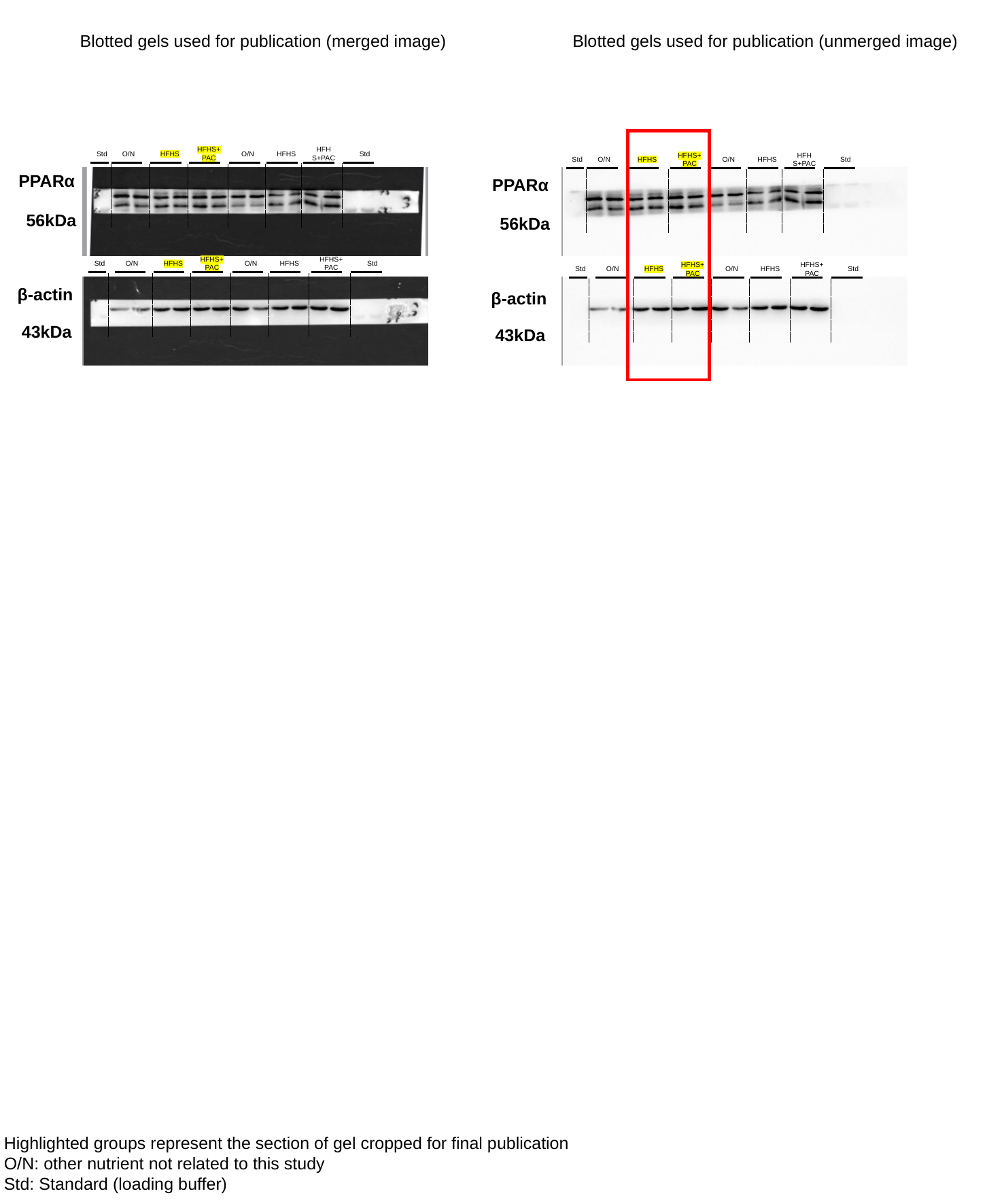

Blotted gels used for publication (merged image)
Blotted gels used for publication (unmerged image)
HFHS+PAC
HFH S+PAC
Std
Std
O/N
HFHS
O/N
HFHS
HFHS+PAC
HFHS+PAC
Std
O/N
HFHS
O/N
HFHS
Std
HFHS+PAC
HFH S+PAC
Std
Std
O/N
HFHS
O/N
HFHS
PPARα
56kDa
β-actin
43kDa
PPARα
56kDa
β-actin
43kDa
HFHS+PAC
HFHS+PAC
Std
O/N
HFHS
O/N
HFHS
Std
Highlighted groups represent the section of gel cropped for final publication
O/N: other nutrient not related to this study
Std: Standard (loading buffer)

## Slide 7
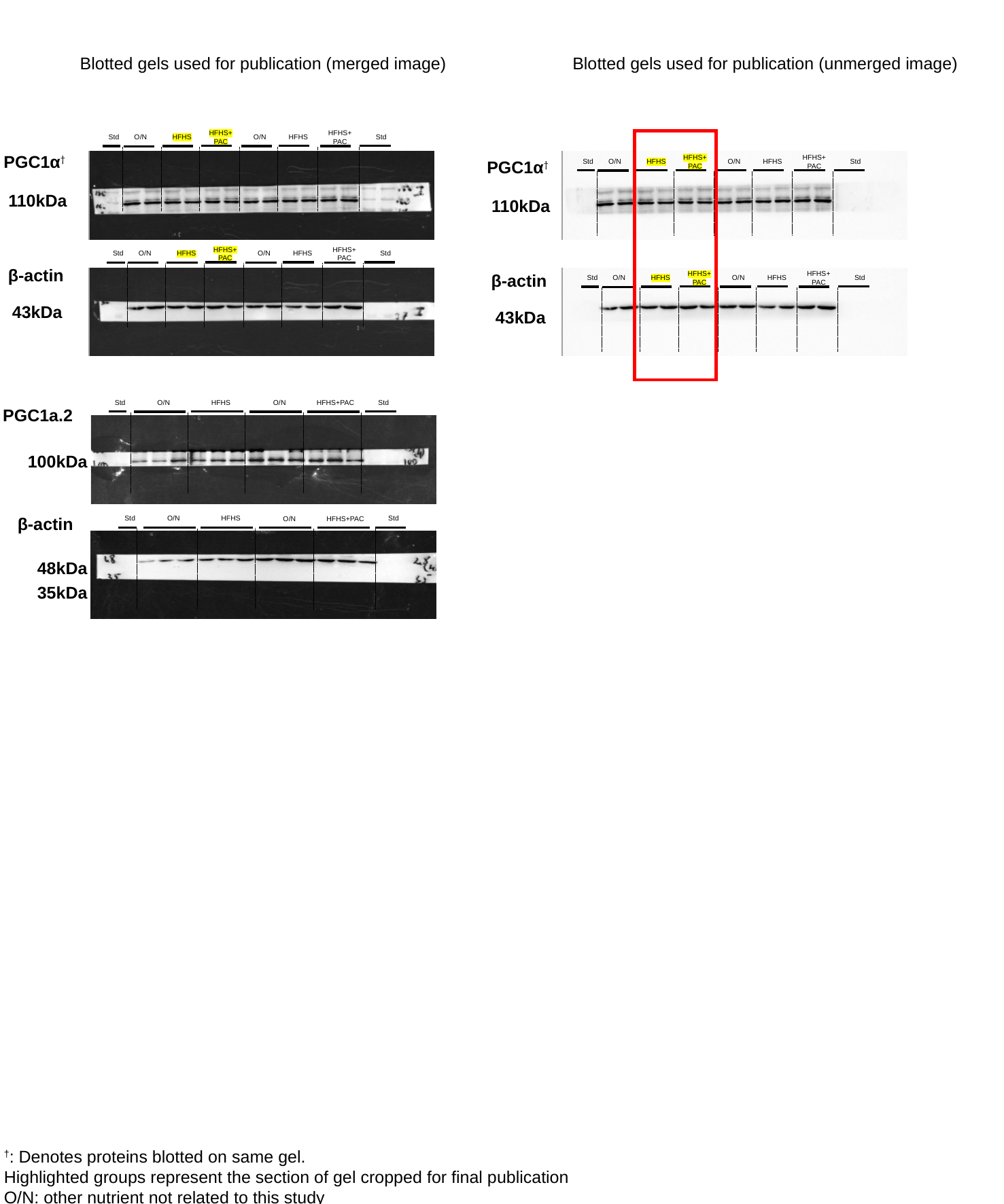

Blotted gels used for publication (merged image)
Blotted gels used for publication (unmerged image)
HFHS+PAC
HFHS+PAC
Std
O/N
HFHS
O/N
HFHS
Std
PGC1α†
110kDa
β-actin
43kDa
HFHS+PAC
HFHS+PAC
Std
O/N
HFHS
O/N
HFHS
Std
PGC1α†
110kDa
β-actin
43kDa
HFHS+PAC
HFHS+PAC
Std
O/N
HFHS
O/N
HFHS
Std
HFHS+PAC
HFHS+PAC
Std
O/N
HFHS
O/N
HFHS
Std
HFHS
Std
O/N
Std
O/N
HFHS+PAC
PGC1a.2
100kDa
β-actin
HFHS
Std
O/N
Std
O/N
HFHS+PAC
48kDa
35kDa
†: Denotes proteins blotted on same gel.
Highlighted groups represent the section of gel cropped for final publication
O/N: other nutrient not related to this study
Std: Standard (loading buffer)

## Slide 8
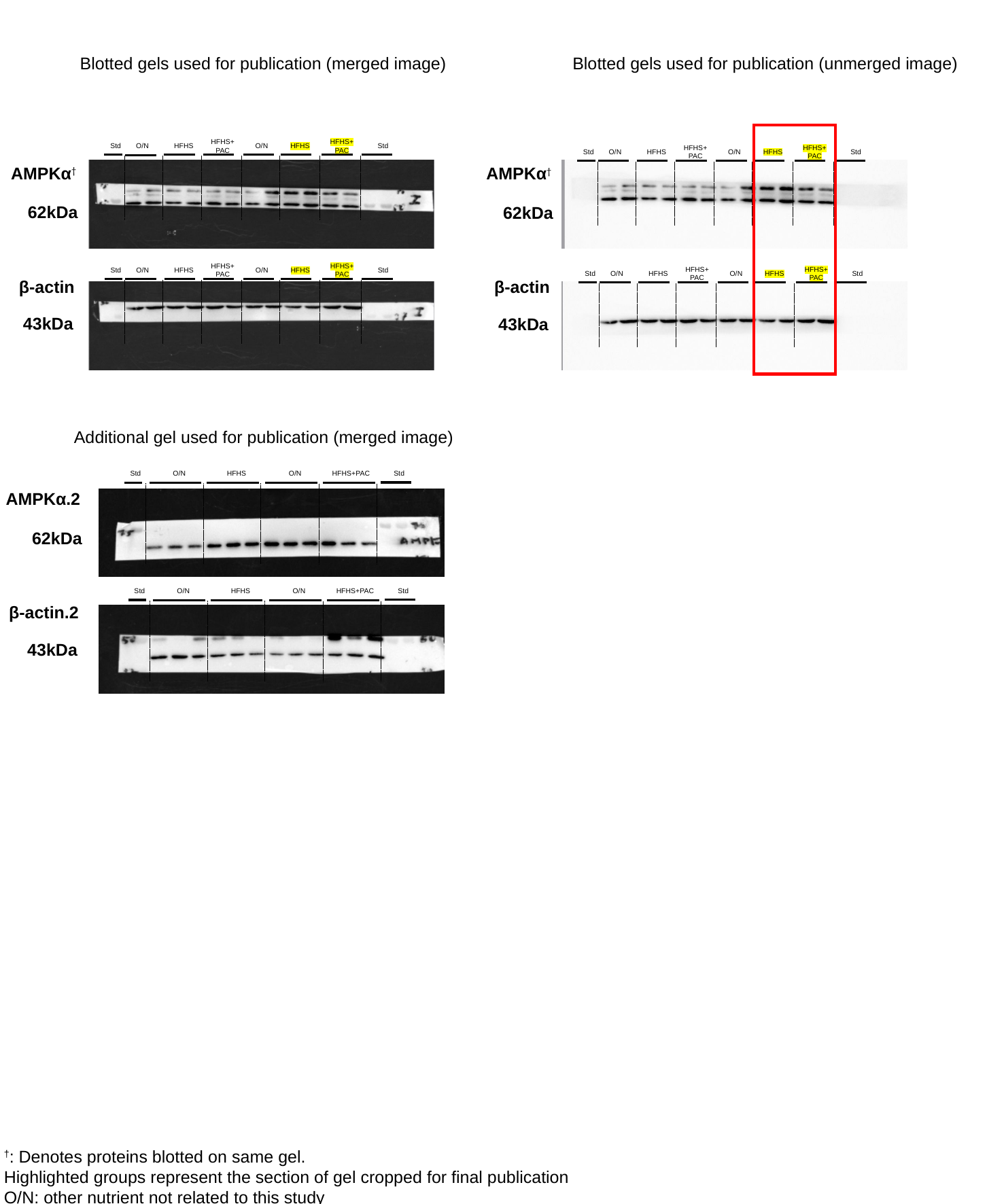

Blotted gels used for publication (merged image)
Blotted gels used for publication (unmerged image)
HFHS+PAC
HFHS+PAC
Std
O/N
HFHS
O/N
HFHS
Std
HFHS+PAC
HFHS+PAC
Std
O/N
HFHS
O/N
HFHS
Std
AMPKα†
62kDa
β-actin
43kDa
AMPKα†
62kDa
β-actin
43kDa
HFHS+PAC
HFHS+PAC
Std
O/N
HFHS
O/N
HFHS
Std
HFHS+PAC
HFHS+PAC
Std
O/N
HFHS
O/N
HFHS
Std
Additional gel used for publication (merged image)
HFHS
Std
O/N
Std
O/N
HFHS+PAC
HFHS
Std
O/N
Std
O/N
HFHS+PAC
AMPKα.2
62kDa
β-actin.2
43kDa
†: Denotes proteins blotted on same gel.
Highlighted groups represent the section of gel cropped for final publication
O/N: other nutrient not related to this study
Std: Standard (loading buffer)

## Slide 9
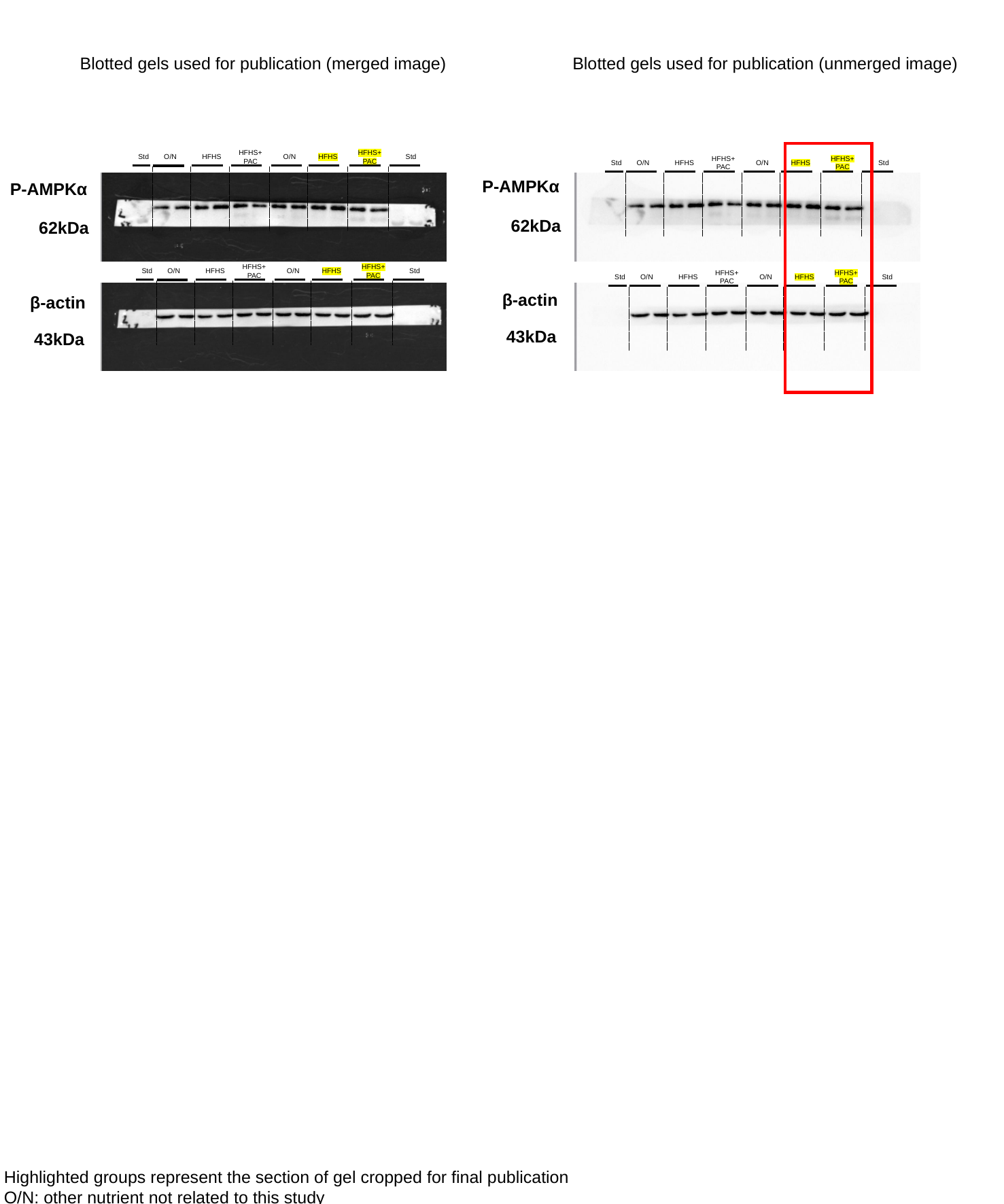

Blotted gels used for publication (merged image)
Blotted gels used for publication (unmerged image)
HFHS+PAC
HFHS+PAC
Std
O/N
HFHS
O/N
HFHS
Std
HFHS+PAC
HFHS+PAC
Std
O/N
HFHS
O/N
HFHS
Std
P-AMPKα
62kDa
β-actin
43kDa
P-AMPKα
62kDa
β-actin
43kDa
HFHS+PAC
HFHS+PAC
Std
O/N
HFHS
O/N
HFHS
Std
HFHS+PAC
HFHS+PAC
Std
O/N
HFHS
O/N
HFHS
Std
Highlighted groups represent the section of gel cropped for final publication
O/N: other nutrient not related to this study
Std: Standard (loading buffer)

## Slide 10
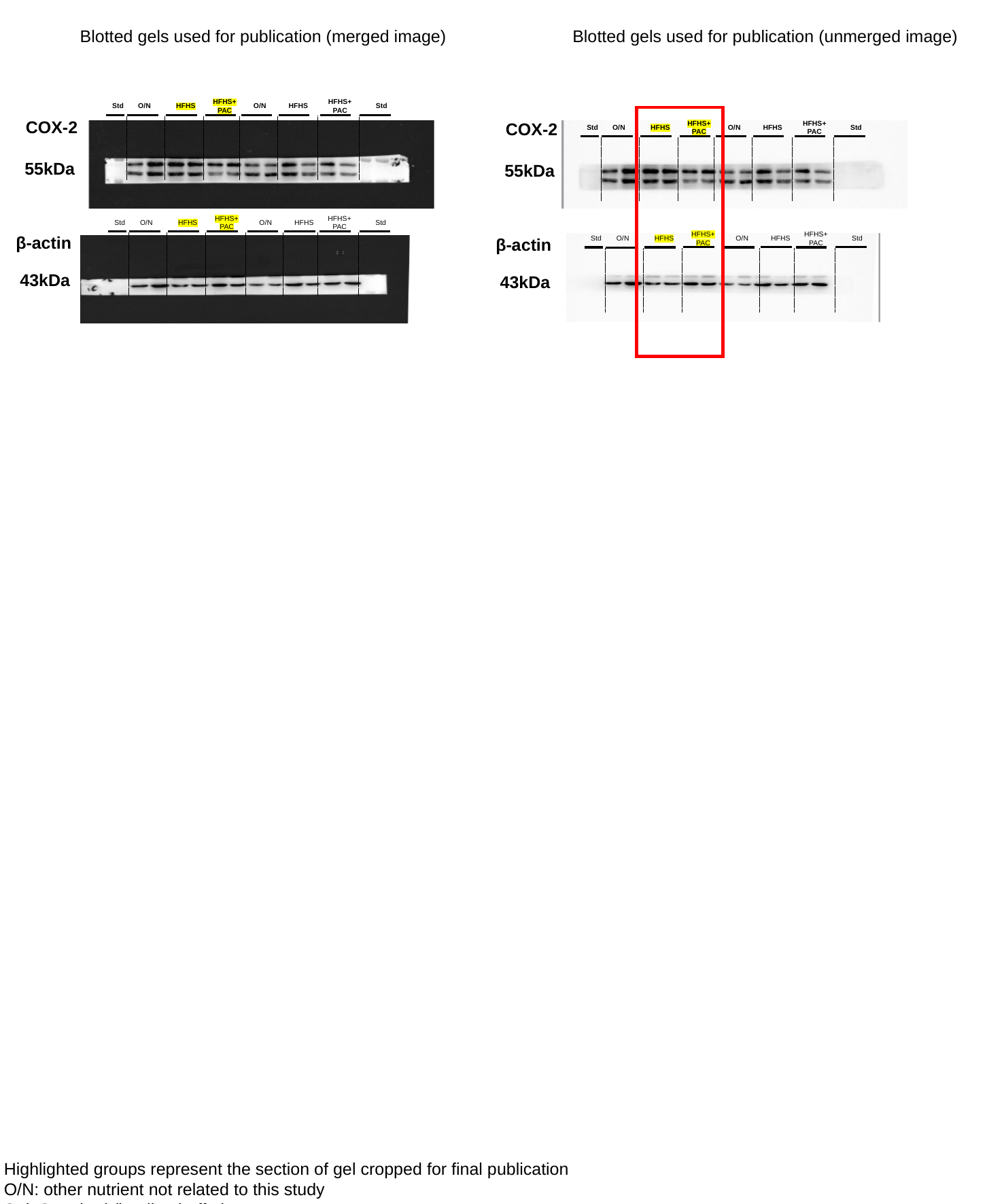

Blotted gels used for publication (merged image)
Blotted gels used for publication (unmerged image)
HFHS+PAC
Std
O/N
HFHS
HFHS+PAC
HFHS
Std
O/N
COX-2
55kDa
β-actin
43kDa
COX-2
55kDa
β-actin
43kDa
HFHS+PAC
Std
O/N
HFHS
HFHS+PAC
HFHS
Std
O/N
HFHS+PAC
HFHS+PAC
Std
O/N
HFHS
O/N
HFHS
Std
HFHS+PAC
HFHS+PAC
Std
O/N
HFHS
O/N
HFHS
Std
Highlighted groups represent the section of gel cropped for final publication
O/N: other nutrient not related to this study
Std: Standard (loading buffer)

## Slide 11
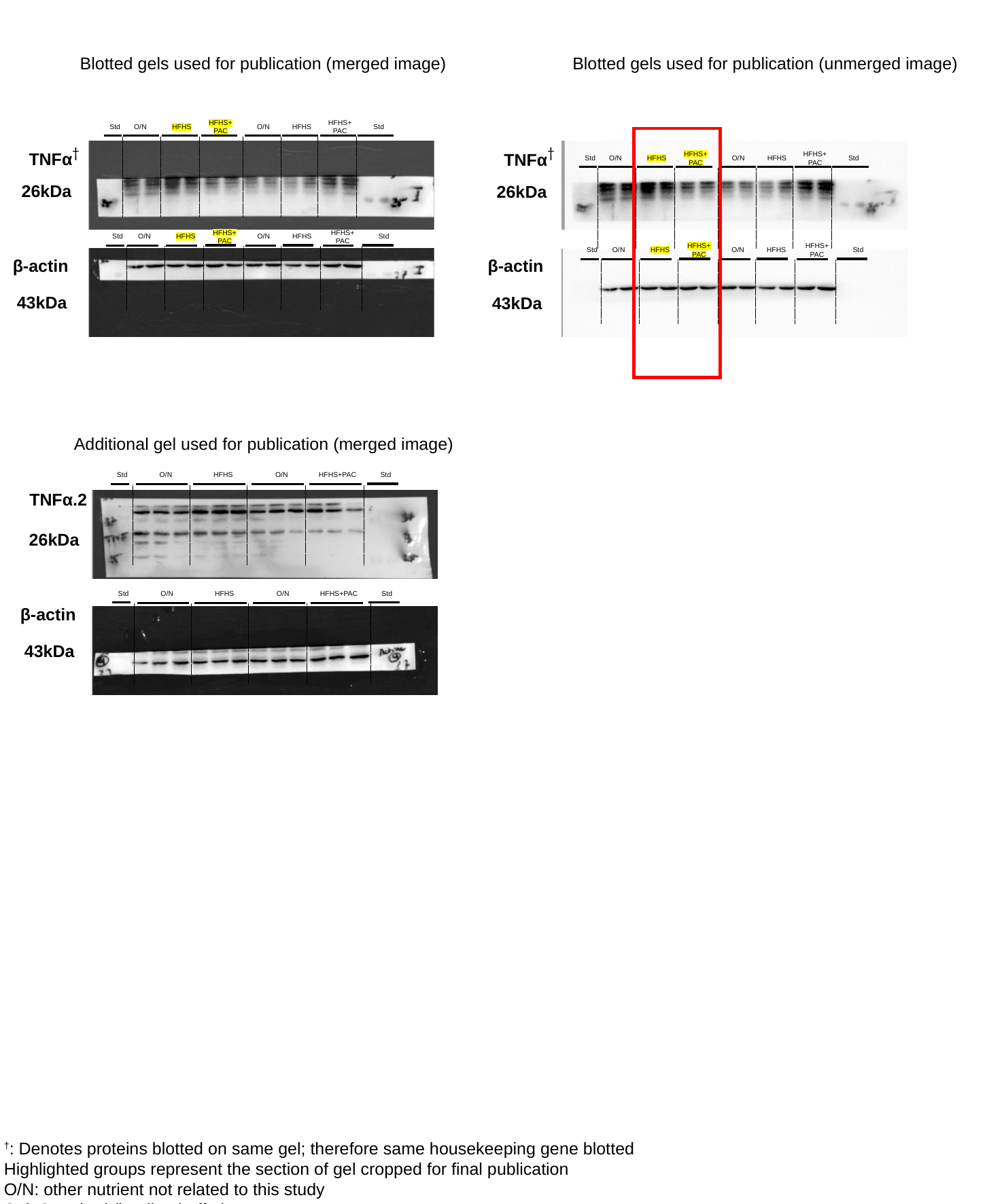

Blotted gels used for publication (merged image)
Blotted gels used for publication (unmerged image)
HFHS+PAC
HFHS+PAC
O/N
HFHS
Std
Std
O/N
HFHS
TNFα†
26kDa
β-actin
43kDa
TNFα†
26kDa
β-actin
43kDa
HFHS+PAC
HFHS+PAC
O/N
HFHS
Std
Std
O/N
HFHS
HFHS+PAC
Std
O/N
HFHS
O/N
HFHS
HFHS+PAC
Std
HFHS+PAC
Std
O/N
HFHS
O/N
HFHS
HFHS+PAC
Std
Additional gel used for publication (merged image)
HFHS
Std
O/N
Std
O/N
HFHS+PAC
TNFα.2
26kDa
β-actin
43kDa
HFHS
Std
O/N
Std
O/N
HFHS+PAC
†: Denotes proteins blotted on same gel; therefore same housekeeping gene blotted
Highlighted groups represent the section of gel cropped for final publication
O/N: other nutrient not related to this study
Std: Standard (loading buffer)

## Slide 12
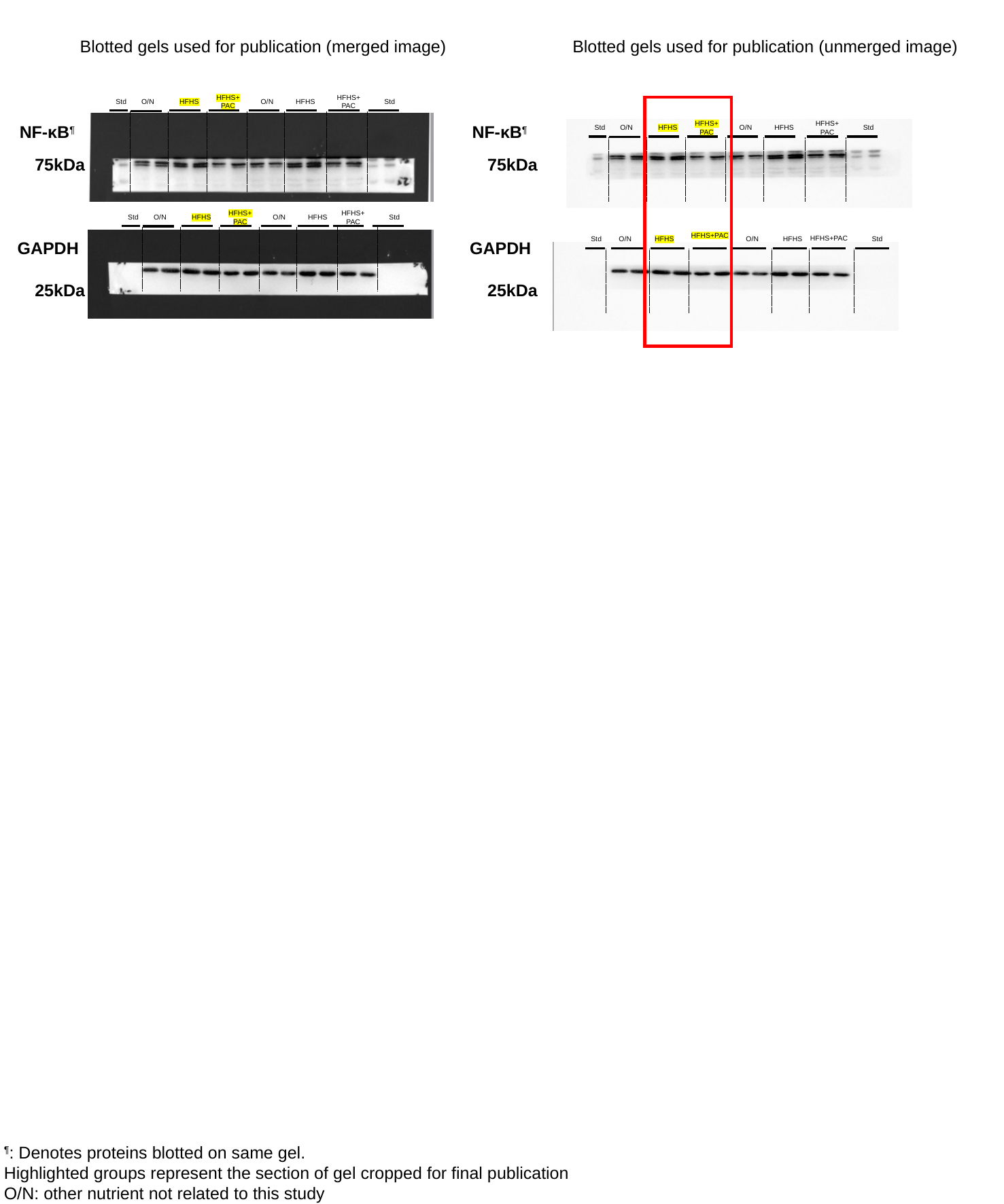

Blotted gels used for publication (merged image)
Blotted gels used for publication (unmerged image)
HFHS+PAC
Std
O/N
HFHS
O/N
HFHS
HFHS+PAC
Std
HFHS+PAC
Std
O/N
HFHS
O/N
HFHS
HFHS+PAC
Std
NF-κB¶
75kDa
GAPDH
25kDa
HFHS+PAC
HFHS+PAC
O/N
HFHS
Std
Std
O/N
HFHS
NF-κB¶
75kDa
GAPDH
25kDa
HFHS+PAC
HFHS+PAC
Std
O/N
HFHS
O/N
HFHS
Std
¶: Denotes proteins blotted on same gel.
Highlighted groups represent the section of gel cropped for final publication
O/N: other nutrient not related to this study
Std: Standard (loading buffer)

## Slide 13
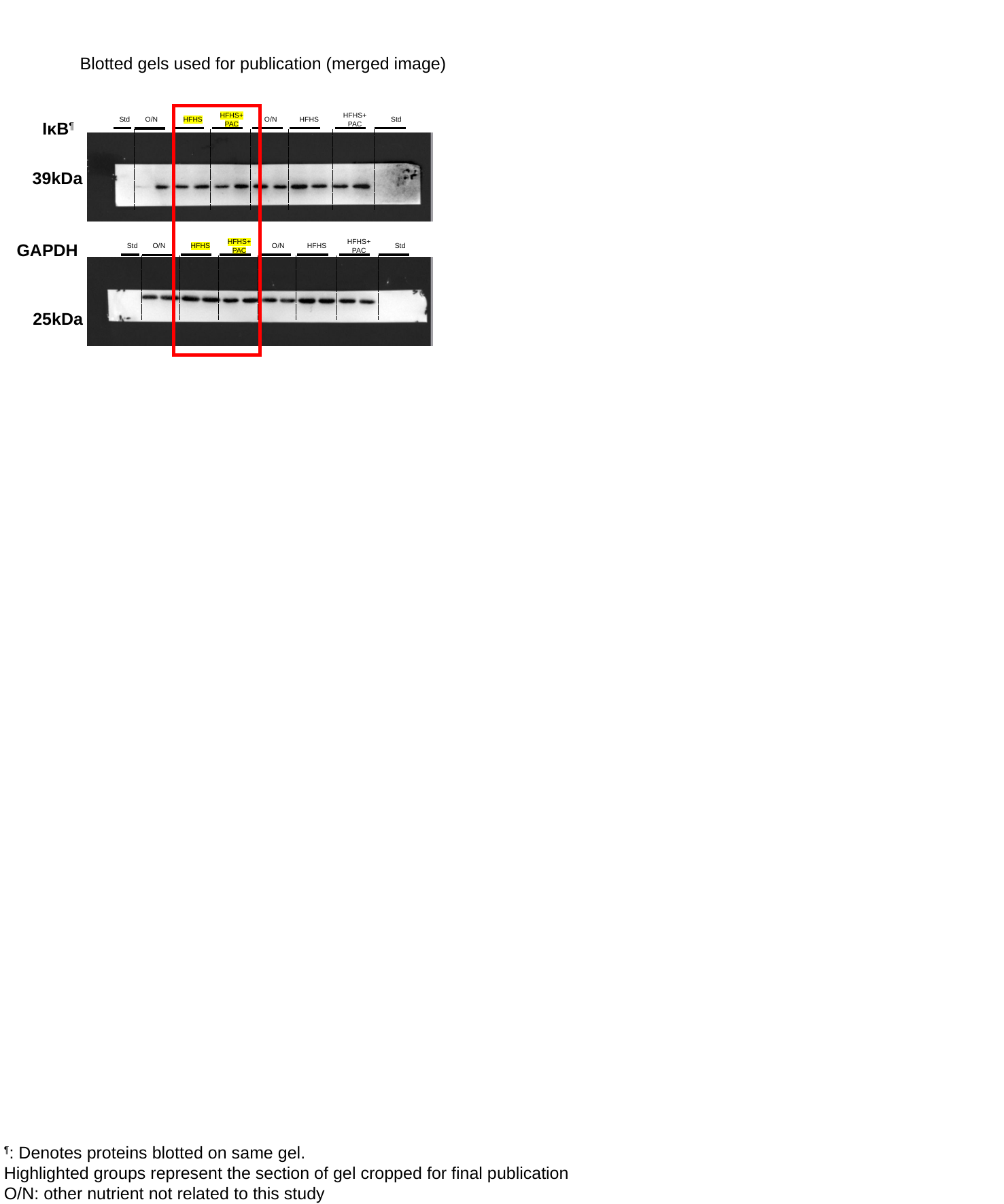

Blotted gels used for publication (merged image)
HFHS+PAC
HFHS+PAC
Std
Std
O/N
HFHS
O/N
HFHS
IκB¶
39kDa
HFHS+PAC
HFHS+PAC
Std
GAPDH
Std
O/N
HFHS
O/N
HFHS
25kDa
¶: Denotes proteins blotted on same gel.
Highlighted groups represent the section of gel cropped for final publication
O/N: other nutrient not related to this study
Std: Standard (loading buffer)

## Slide 14
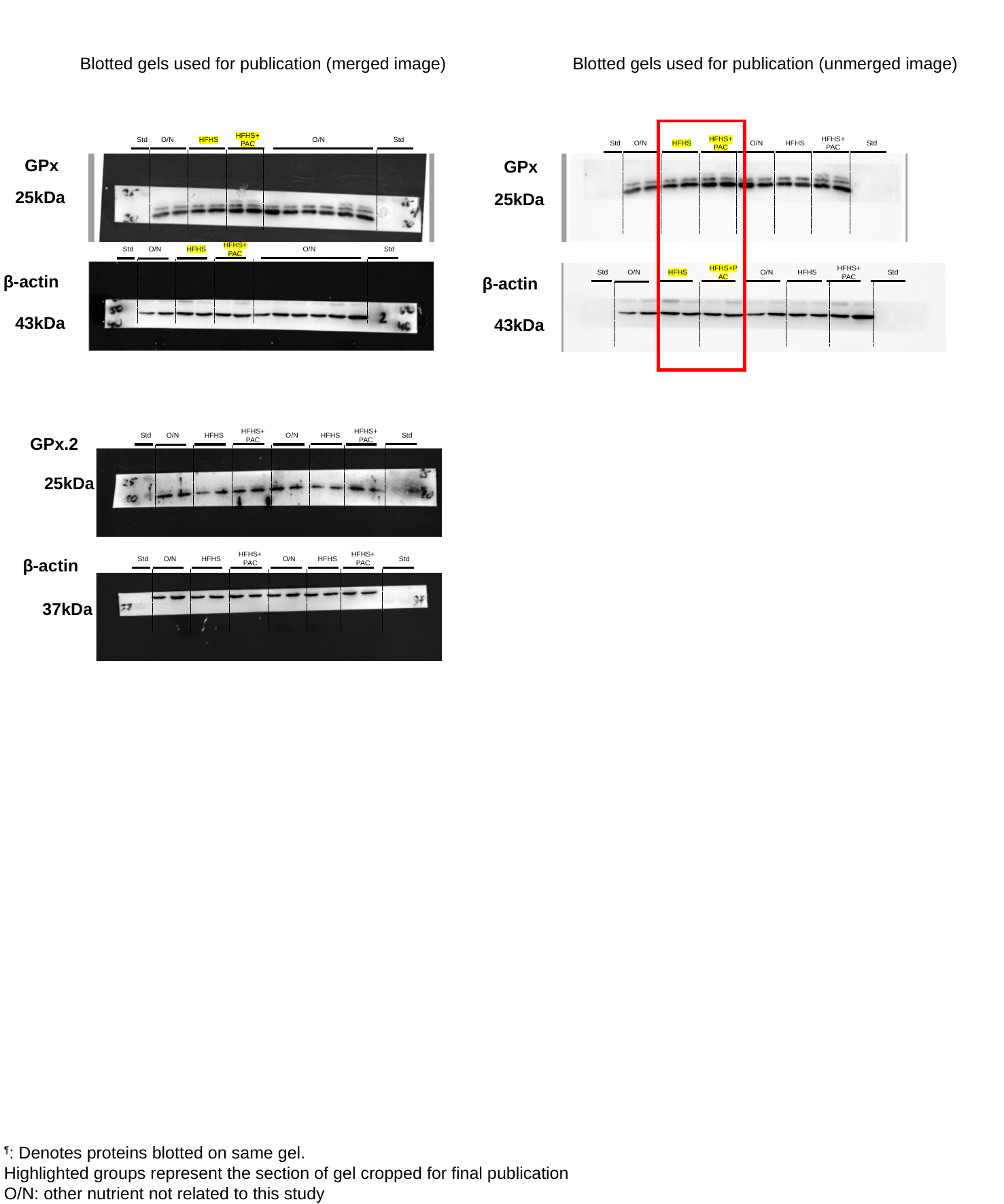

Blotted gels used for publication (merged image)
Blotted gels used for publication (unmerged image)
HFHS+PAC
Std
O/N
HFHS
O/N
Std
HFHS+PAC
HFHS+PAC
O/N
HFHS
Std
Std
O/N
HFHS
GPx
25kDa
β-actin
43kDa
GPx
25kDa
β-actin
43kDa
HFHS+PAC
Std
O/N
HFHS
O/N
Std
HFHS+PAC
HFHS+ PAC
Std
O/N
HFHS
O/N
HFHS
Std
HFHS+PAC
HFHS+PAC
Std
O/N
HFHS
O/N
HFHS
Std
GPx.2
β-actin
25kDa
37kDa
HFHS+PAC
HFHS+PAC
Std
O/N
HFHS
O/N
HFHS
Std
¶: Denotes proteins blotted on same gel.
Highlighted groups represent the section of gel cropped for final publication
O/N: other nutrient not related to this study
Std: Standard (loading buffer)

## Slide 15
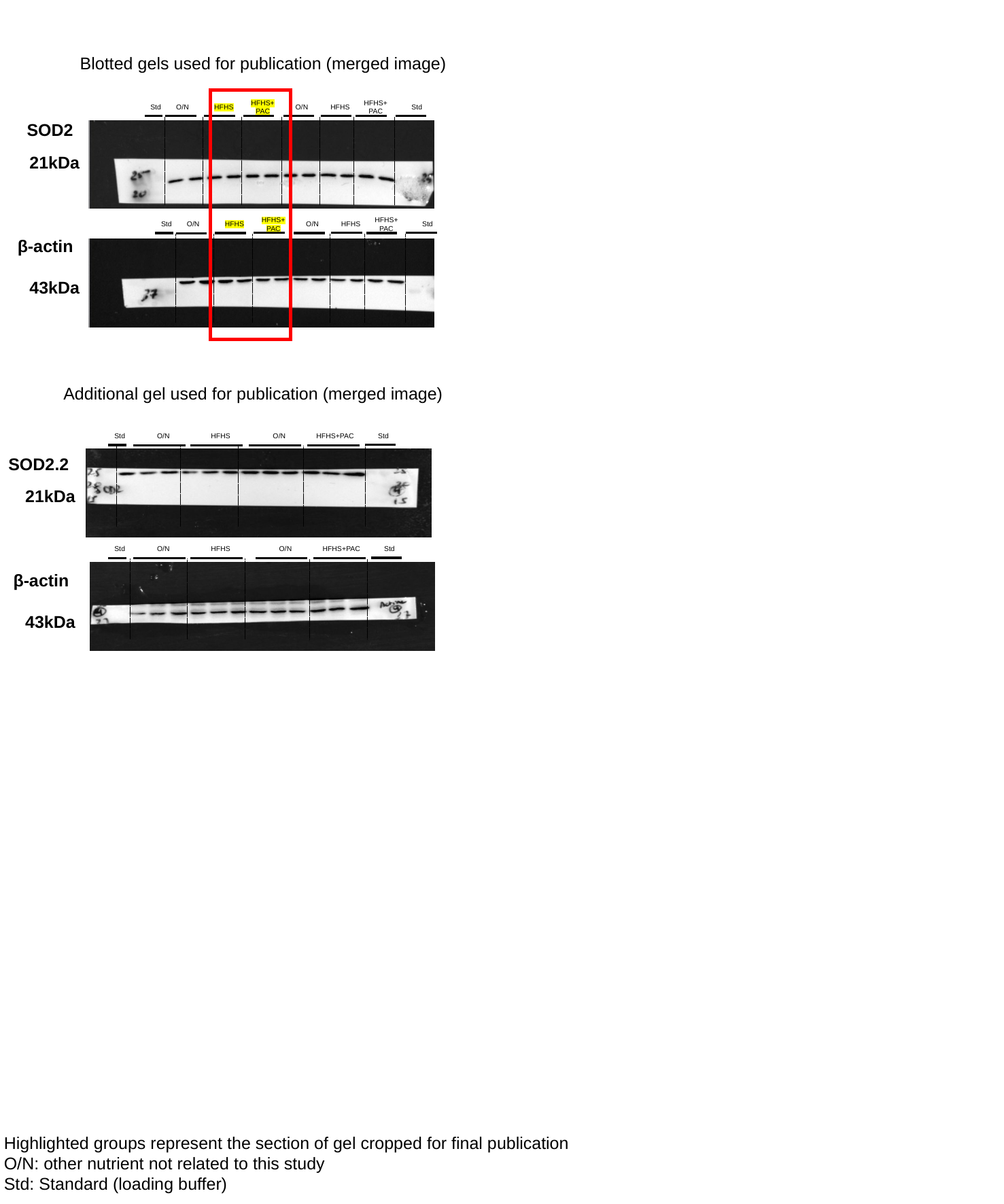

Blotted gels used for publication (merged image)
HFHS+PAC
HFHS+PAC
Std
O/N
HFHS
O/N
HFHS
Std
SOD2
21kDa
β-actin
43kDa
HFHS+PAC
HFHS+PAC
Std
O/N
HFHS
O/N
HFHS
Std
Additional gel used for publication (merged image)
HFHS
Std
O/N
Std
O/N
HFHS+PAC
SOD2.2
21kDa
β-actin
43kDa
HFHS
Std
O/N
Std
O/N
HFHS+PAC
Highlighted groups represent the section of gel cropped for final publication
O/N: other nutrient not related to this study
Std: Standard (loading buffer)

## Slide 16
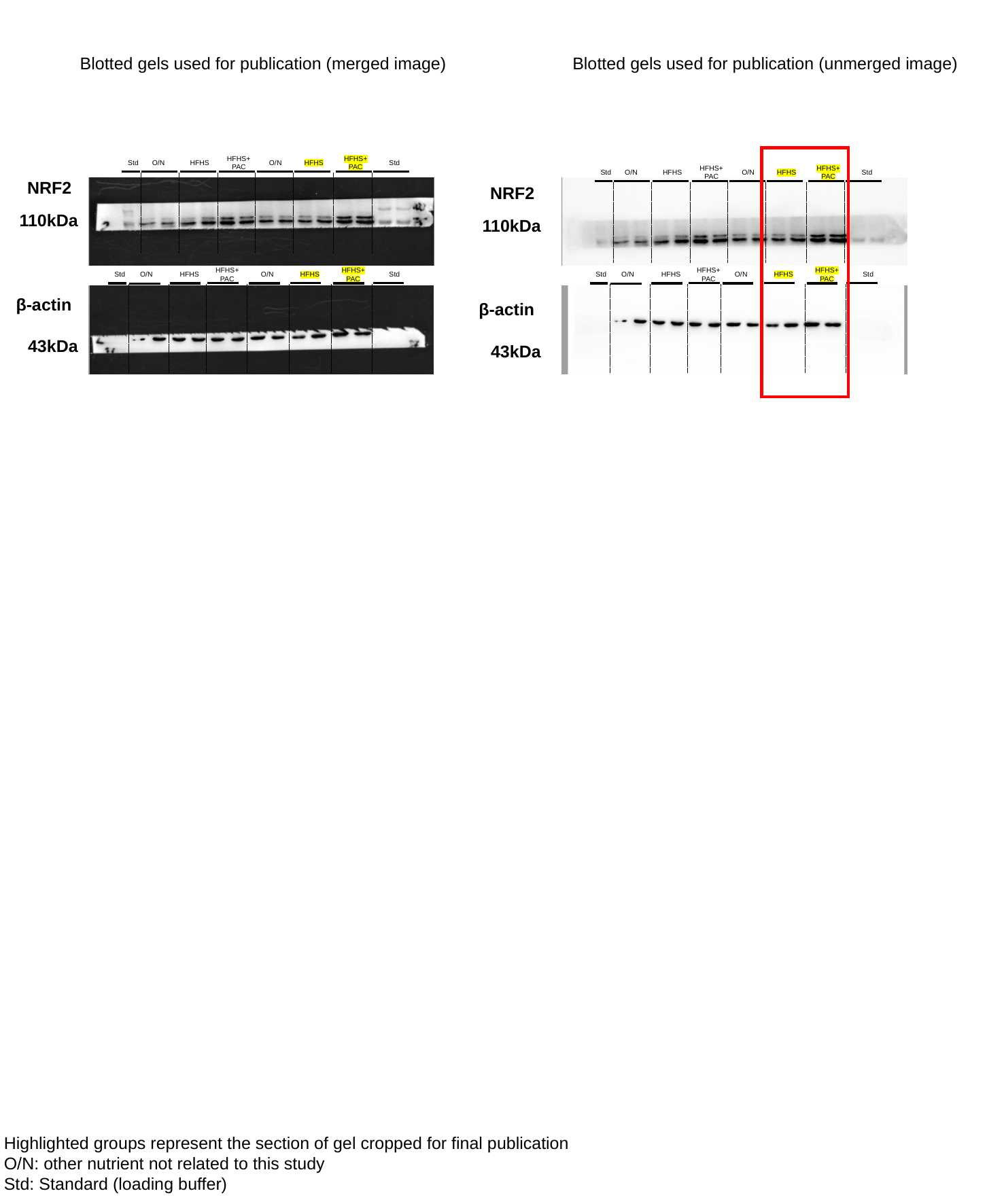

Blotted gels used for publication (merged image)
Blotted gels used for publication (unmerged image)
HFHS+PAC
HFHS+PAC
Std
Std
O/N
HFHS
O/N
HFHS
HFHS+PAC
HFHS+PAC
Std
Std
O/N
HFHS
O/N
HFHS
NRF2
110kDa
β-actin
43kDa
NRF2
110kDa
β-actin
43kDa
HFHS+PAC
HFHS+PAC
O/N
HFHS
Std
HFHS+PAC
HFHS+PAC
Std
O/N
HFHS
O/N
HFHS
Std
Std
O/N
HFHS
Highlighted groups represent the section of gel cropped for final publication
O/N: other nutrient not related to this study
Std: Standard (loading buffer)

## Slide 17
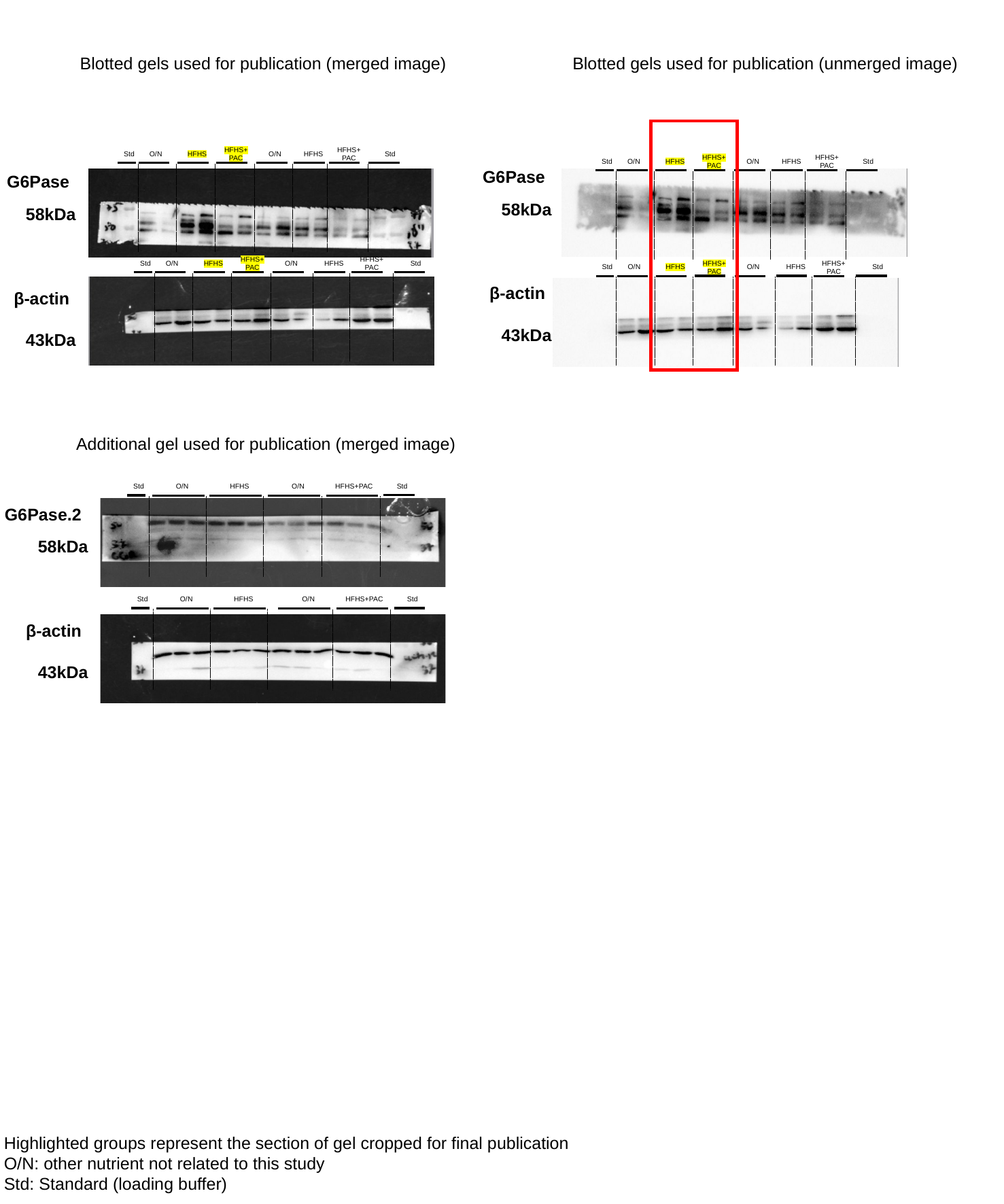

Blotted gels used for publication (merged image)
Blotted gels used for publication (unmerged image)
HFHS+PAC
HFHS+PAC
Std
O/N
HFHS
O/N
HFHS
Std
HFHS+PAC
HFHS+PAC
Std
O/N
HFHS
O/N
HFHS
Std
G6Pase
58kDa
β-actin
43kDa
G6Pase
58kDa
β-actin
43kDa
HFHS+PAC
HFHS+PAC
Std
O/N
HFHS
O/N
HFHS
Std
HFHS+PAC
HFHS+PAC
Std
O/N
HFHS
O/N
HFHS
Std
Additional gel used for publication (merged image)
HFHS
Std
O/N
Std
O/N
HFHS+PAC
G6Pase.2
58kDa
β-actin
43kDa
HFHS
Std
O/N
Std
O/N
HFHS+PAC
Highlighted groups represent the section of gel cropped for final publication
O/N: other nutrient not related to this study
Std: Standard (loading buffer)

## Slide 18
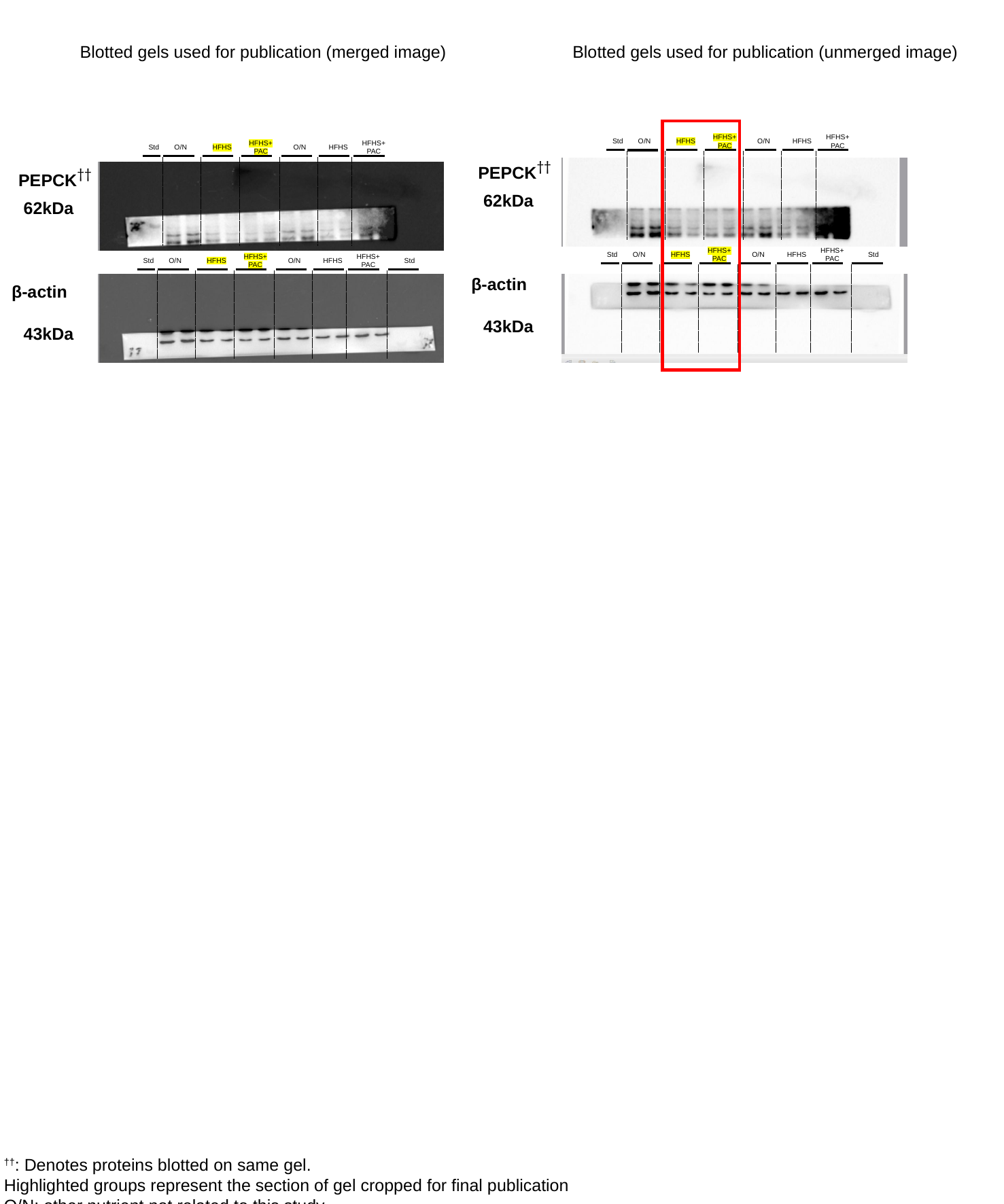

Blotted gels used for publication (merged image)
Blotted gels used for publication (unmerged image)
HFHS+PAC
HFHS+PAC
Std
O/N
HFHS
O/N
HFHS
HFHS+PAC
HFHS+PAC
Std
O/N
HFHS
O/N
HFHS
PEPCK††
62kDa
β-actin
43kDa
PEPCK††
62kDa
β-actin
43kDa
HFHS+PAC
HFHS+PAC
Std
O/N
HFHS
O/N
HFHS
Std
HFHS+PAC
HFHS+PAC
Std
O/N
HFHS
O/N
HFHS
Std
††: Denotes proteins blotted on same gel.
Highlighted groups represent the section of gel cropped for final publication
O/N: other nutrient not related to this study
Std: Standard (loading buffer)

## Slide 19
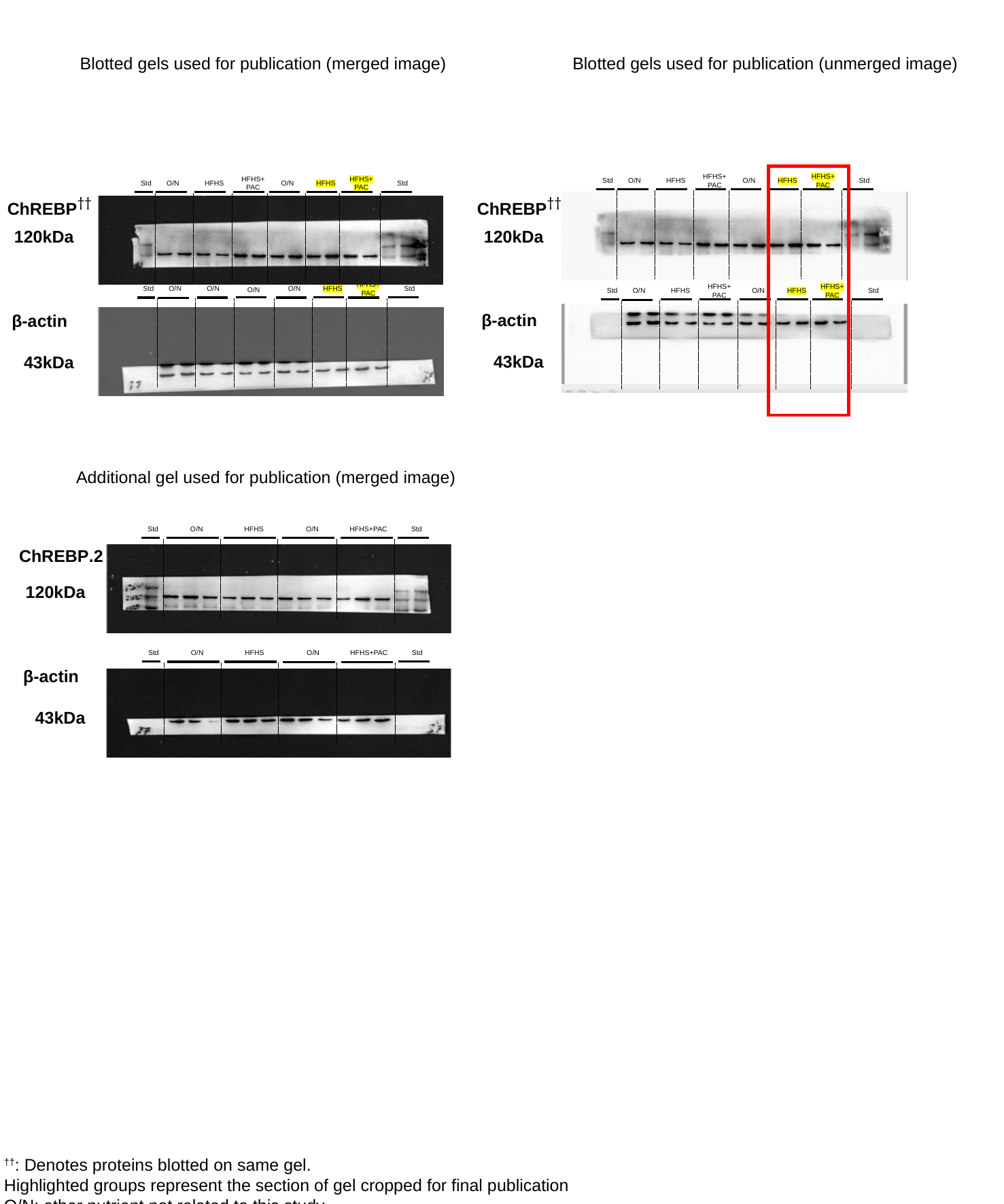

Blotted gels used for publication (merged image)
Blotted gels used for publication (unmerged image)
HFHS+PAC
HFHS+PAC
O/N
HFHS
Std
Std
O/N
HFHS
HFHS+PAC
HFHS+PAC
O/N
HFHS
Std
Std
O/N
HFHS
ChREBP††
120kDa
β-actin
43kDa
ChREBP††
120kDa
β-actin
43kDa
HFHS+PAC
Std
O/N
O/N
O/N
HFHS
Std
O/N
HFHS+PAC
HFHS+PAC
Std
O/N
HFHS
O/N
HFHS
Std
Additional gel used for publication (merged image)
HFHS
Std
O/N
Std
O/N
HFHS+PAC
ChREBP.2
120kDa
β-actin
43kDa
HFHS
Std
O/N
Std
O/N
HFHS+PAC
††: Denotes proteins blotted on same gel.
Highlighted groups represent the section of gel cropped for final publication
O/N: other nutrient not related to this study
Std: Standard (loading buffer)
